# Ultra-Wideband Diversity MIMO Antenna System for Future Mobile Handsets

**DOI:** 10.3390/s20082371

**Published:** 2020-04-22

**Authors:** Naser Ojaroudi Parchin, Haleh Jahanbakhsh Basherlou, Yasir I. A. Al-Yasir, Ahmed M. Abdulkhaleq, Raed A. Abd-Alhameed

**Affiliations:** 1 Faculty of Engineering and Informatics, University of Bradford, Bradford BD7 1DP, UK; Y.I.A.Al-Yasir@bradford.ac.uk (Y.I.A.A.-Y.); A.Abd@sarastech.co.uk (A.M.A.); R.A.A.Abd@bradford.ac.uk (R.A.A.-A.); 2 Bradford College, West Yorkshire, Bradford BD7 1AY, UK; Hale.Jahanbakhsh@gmail.com; 3 SARAS Technology Limited, Leeds LS12 4NQ, UK; 4 Department of Communication and Informatics Engineering, Basra University College of Science and Technology, Basra 61004, Iraq

**Keywords:** double-fed slot antenna, MIMO system, mobile terminals, polarization diversity, UWB technology

## Abstract

A new ultra-wideband (UWB) multiple-input/multiple-output (MIMO) antenna system is proposed for future smartphones. The structure of the design comprises four identical pairs of compact microstrip-fed slot antennas with polarization diversity function that are placed symmetrically at different edge corners of the smartphone mainboard. Each antenna pair consists of an open-ended circular-ring slot radiator fed by two independently semi-arc-shaped microstrip-feeding lines exhibiting the polarization diversity characteristic. Therefore, in total, the proposed smartphone antenna design contains four horizontally-polarized and four vertically-polarized elements. The characteristics of the single-element dual-polarized UWB antenna and the proposed UWB-MIMO smartphone antenna are examined while using both experimental and simulated results. An impedance bandwidth of 2.5–10.2 GHz with 121% fractional bandwidth (FBW) is achieved for each element. However, for S_11_ ≤ −6 dB, this value is more than 130% (2.2–11 GHz). The proposed UWB-MIMO smartphone antenna system offers good isolation, dual-polarized function, full radiation coverage, and sufficient efficiency. Besides, the calculated diversity performances of the design in terms of the envelope correlation coefficient (ECC) and total active reflection coefficient (TARC) are very low over the entire operating band.

## 1. Introduction

Ultra-Wideband (UWB) technology provides some superiority, such as high-speed data transmission, low cost, and easy manufacture [1]. However, such a popular topic of technology suffers from multipath fading in practical applications [2]. Precisely, the multiple-input multiple-output (MIMO) technique was raised to resolve this issue [3,4]. The combination of UWB and MIMO technologies, the use of space multipath, parallel transmission of multiple signals, can obtain obvious multiplexing gain and diversity gain, and achieve a stable signal transmission in distance [5,6]. UWB technology provides wide bandwidth with low power usage. Unlike narrowband technologies, like Bluetooth and Wi-Fi, this technology might be more suitable for short-distance communications [7]. However, it is natively more precise, uses less power, and can transmit data over a wider frequency (up to several GHz). A precise angle combined with a precise distance, which leads the ability for a mobile handset to pinpoint an object to a reasonably precise location in space, as well as to recognize its surroundings. While the UWB technology is not new, its first implementation into a modern smartphone has been used by the world’s smartphone makers, such as Apple [8]. It should be noted that other technologies, like Bluetooth and Wi-Fi, are still useful since they have a longer range. 

Through MIMO technology with multiple antennas, the UWB can measure the angle of arrivals for the signals, as well as secure access to a myriad of the system [9]. Standard MIMO systems tend to employ two or four elements in a single physical package. However, a high number of antenna radiators are employed for the massive MIMO system [10,11]. When compared with previous generations, a large number of antenna elements, operating concurrently, is expected to be applied for 5G communications. 2 × 2 MIMO systems are successfully deployed for 4G cellular networks, while, for the massive MIMO operation of 5G communications, it is expected to employ a large number of elements, since the greater number makes the system more resistant to interferences [12,13]. Therefore, the multi-antenna structure with minimum mutual coupling employed for generating polarization and pattern diversity for reliable communication in order to load the MIMO operation into a smartphone for 5G communication. The 5G network also needs fundamental technologies to enable small cells, beamforming, full duplexing, MIMO, and millimeter-wave (MM-Wave) [14,15]. Simple structured and compact antenna elements with sufficient impedance bandwidth and isolation are desirable to be integrated into 5G smartphone platforms, in accordance with the requirement of mobile networks [16,17,18]. Among various MIMO antennas, microstrip antenna elements are more applicable due to their promising features, such as compact-size, Omani-directional radiation, broad impedance bandwidth, ease of integration, and manufacturing [19,20]. 

Several MIMO 5G smartphone antennas have been recently proposed [21,22,23,24,25,26,27,28,29,30,31,32,33,34,35,36,37,38]. However, these smartphone antennas either suffer from narrow impedance-bandwidth (either single-band or dual/multi-band) or use single-polarized uniplanar antenna resonators occupying large spaces of the mainboard, which leads to increasing the complexity of the mobile phone system. In addition, in most of the reported antennas, all of the candidate bands of sub 6 GHz 5G, including LTE Band 7 (2.550–2.650 GHz), N77 (3.3–4.2 GHz), N78 (3.3–3.8 GHz), N79 (4.4–5.0 GHz), and LTE band 46 (5150–5925 MHz) are not supported. Additionally, in the designs of many reported MIMO smartphone antennas, it is common to avoid placing elements in vertical and choose instead to place them parallel, which prevents achieving the polarization diversity. However, in this paper, the antenna elements are both perpendicular and parallel to each other to exhibit the diversity function. In [21,22], uniplanar and double-layer antenna elements with only 200 MHz bandwidths are proposed for 5G smartphone applications. Tightly arranged orthogonal-mode pairs with design complexity is proposed in [23] for 5G smartphones. In [24,25], CPW-fed antenna arrays with large-clearance are introduced for sub 6 GHz MIMO handset. In [26,27,28], narrow-band antenna arrays with 100 MHz are represented to be integrated at the corners of the mainboard. The proposed antennas in [29,30] use slot resonators in the ground plane, providing polarization diversity function at 3.6 GHz. A multi-element multi-layer antenna with the operation band of 4.4–4.7 GHz is proposed in [31]. The proposed antennas in [32,33,34] have uniplanar configurations which make them difficult to fabricate and integrate with the circuit system. In addition, the proposed smartphone antenna array in [35] with wide impedance bandwidth employing only two radiators, which is not sufficient for 5G MIMO operation. Moreover, broadband MIMO arrays with 2 GHz bandwidths are reported in [36,37]. The employed radiators are not planar and careful consideration is required in the fabrication process. In [38], an eight-element PIFA array arranged on an artificial magnetic plane with less possible screen-space is proposed.

In this manuscript, we present a new design of broadband MIMO antenna systems providing polarization diversity and ultra-wide impedance bandwidth. To the best of our knowledge, this is the first integration of multi UWB antennas with polarization and pattern diversity onto a MIMO smartphone system. The smartphone antenna is designed to work at 2.5–10.2 GHz, covering different wireless systems, including LTE-4G, sub 6 GHz 5G, WLAN, WIMAX, X-band, etc. The structure of the smartphone MIMO array design comprises four pairs of orthogonally dual-polarized slot radiators located at four edge corners of the smartphone PCB. By exciting the radiator from the different feeding ports, two orthogonally polarized waves are generated, which leads the radiation pattern and polarization diversity function. A low-cost FR-4 dielectric is used as the PCB substrate. A prototype sample of the proposed design was fabricated and then tested. Good impedance bandwidth and isolation are achieved. The characteristics of the single radiator and its MIMO package are elaborated below.

## 2. Dual-Polarized UWB Slot Antenna

**Figure 1:**
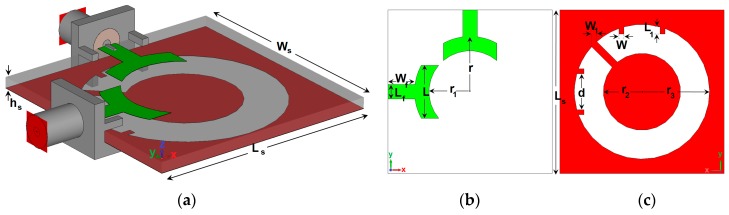
Overall view of the single-element Ultra-Wideband (UWB) antenna design, (**a**) three-dimensional (3D) view, (**b**) top, and (**c**) bottom layers.

Figure 1 provides the geometry of the designed UWB diversity antenna design. As shown, the design is composed of an open-ended circular-ring slot radiator with outer and inner radiuses of r_3_ and r_2_ differently fed by a pair of symmetrical semi-arc-shaped microstrip-line in orthogonal positions. As shown in Figure 1a, 50-Ohm SMA (SubMiniature version A) connectors are employed for both antenna feeding ports. Table 1 lists the dimensions of the proposed UWB diversity antenna element. The antenna is designed on an FR4 dielectric whose permittivity and loss tangent are 4.4 and 0.025, respectively. The antenna has an overall dimension of W_S_ × L_S_ × h_S_ = 34 × 34 × 1.6 mm^3^. It is operating at the frequency range of 2.5 to 10.2 GHz. The impedance matching band of the proposed slot antenna is governed by several parameters, such as the dimensions of the open-ended circular-ring slot and the semi-arc-shaped radiation stub, as well as the small strip pairs protruded to increase the matching band and mutual coupling, especially at high frequencies. All of these parameters were optimized to enable an UWB operating frequency band with sufficient mutual coupling inferior to −10 dB.

**Figure 2:**
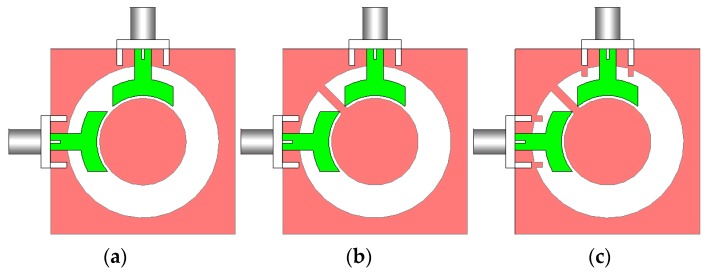
Various structures of the UWB slot antenna, (**a**) antenna with a circular-ring slot radiator, (**b**) the antenna with open-ended circular-ring slot, and (**c**) the proposed UWB antenna design.

**Figure 3:**
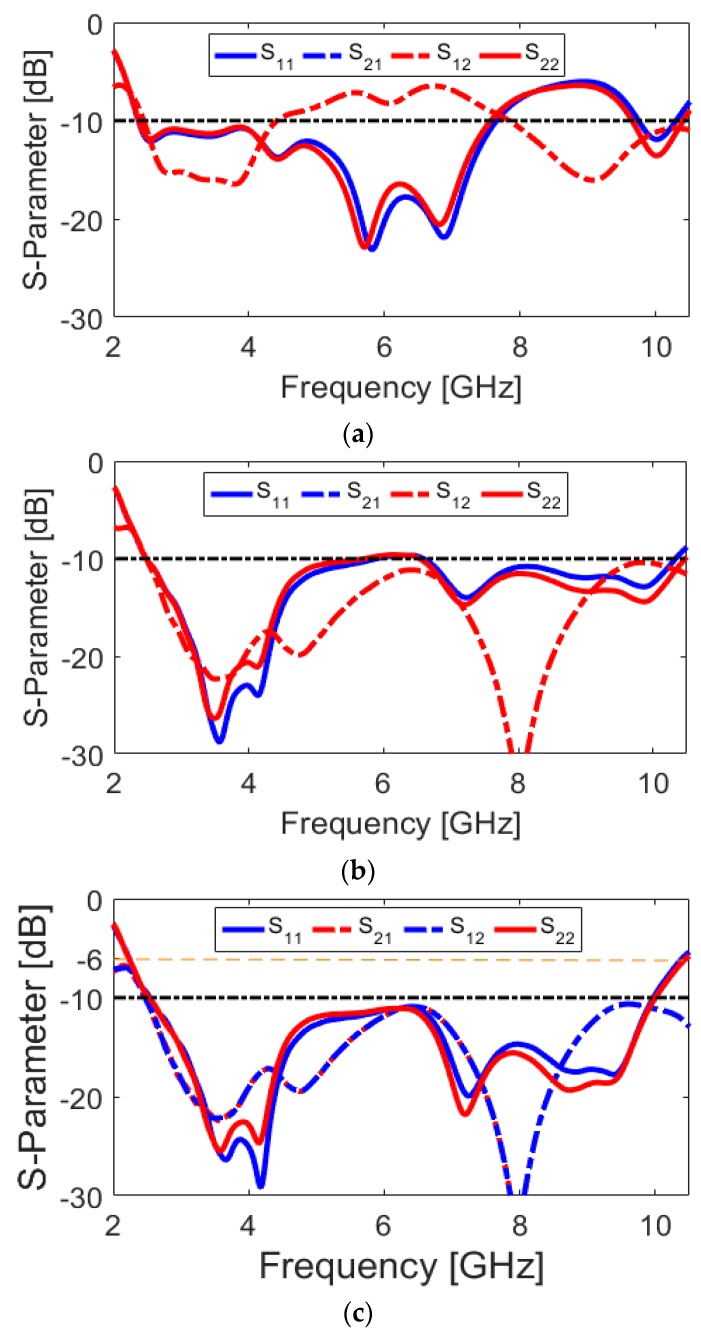
S parameters of the (**a**) antenna with a circular-ring slot radiator, (**b**) the antenna with open-ended circular-ring slot, and (**c**) the proposed UWB antenna design.

Various configurations of the antenna are shown in Figure 2. Simulated S parameter results of the antenna design with a conventional circular-ring slot radiator (Figure 2a), with an open-ended circular-ring slot radiator (Figure 2b), and the presented design (Figure 2c) are shown in Figure 3a–c, respectively. As illustrated, by converting the circular-ring slot radiator into the open-ended circular-ring slot, not only the frequency bandwidth of the antenna but also the isolation characteristic of the design have been significantly improved. Finally, by adding pairs of small strips under the antenna feed-lines, the antenna provides good matching with improved-bandwidth and well-isolated characteristics at the desired frequency band (2.5–10 GHz).

**Figure 4:**
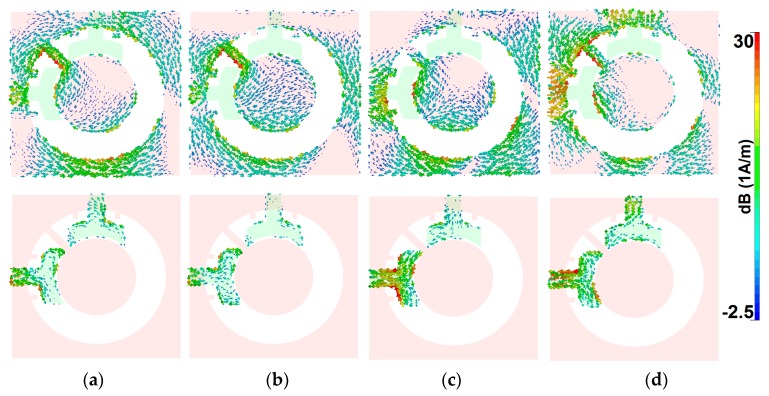
Current densities from Port 1 at resonances: (**a**) 3.8, (**b**) 4.2, (**c**) 7.1, and (**d**) 9.1 GHz.

The current densities of the antenna (port 1) at different resonance frequencies (including 3.8, 4.2, 7.1, and 9.1 GHz) are presented in Figure 4 and discussed in the following. It is obvious that the maximum distributions of the surface current have been mainly concentrated around the ring-slot cut of the ground plane, since it is the main resonator of the design, which provides different resonances of the antenna. Additionally, the semi-arc-shaped microstrip feeding lines current is very active at different frequencies. However, the current is highly concentrated around the edge of the open-ended circular-ring at lower frequencies, as can be observed in Figure 4a,b. This can be explained from Figure 3. It is observed that by converting the circular-ring slot radiator to the open-ended circular-ring slot, the isolation characteristic of the antenna S_11_/S_22_ has been improved significantly (from −12 to less than −25 dB), according to the obtained results from Figure 3a,b. Moreover, the semi-arc-shaped radiation stub and the protruded small rectangular strips in the ground plane appear very active with high current densities at the upper frequencies (7.1 and 9.1 GHz), as shown in Figure 4c,d. This is mainly because of their positive impact on further enhancement of the antenna performance, especially at higher frequencies, as shown in Figure 3c.

**Figure 5:**
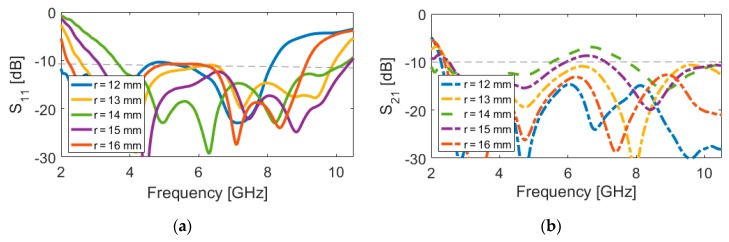
(**a**) S_11_ and (**b**) S_21_ characteristics for different values of r.

**Figure 6:**
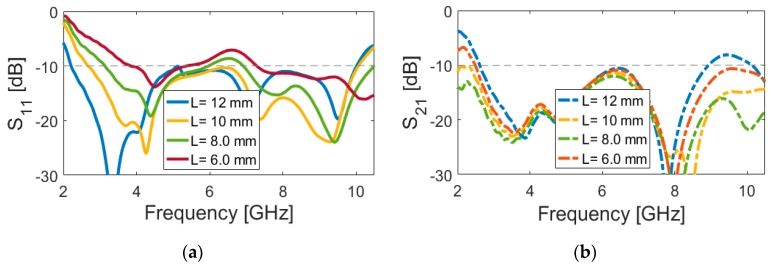
(**a**) S_11_ and (**b**) S_21_ characteristics for different values of (**a**) L.

**Figure 7:**
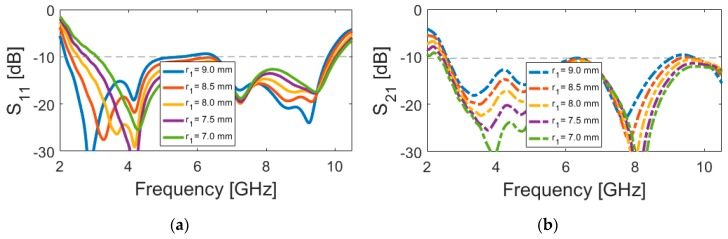
(**a**) S_11_ and **(b**) S_21_ characteristics for different values of r_1_.

**Figure 8:**
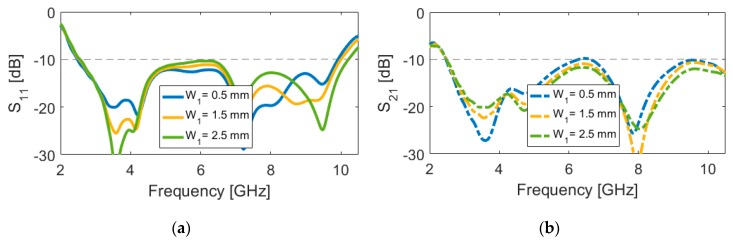
(**a**) S_11_ and (**b**) S_21_ characteristics for different values of W_1_.

The reflection and transmission coefficients (S_11_ and S_21_) of the multi-resonance/UWB-MIMO antenna with various parameters are investigated. Figure 5, Figure 6, Figure 7 and Figure 8 show the results of varying fundamental parameters r, L, r_1_, and W_1_. In the simulation of the designed antenna, when one parameter changes, the rest of the parameters are kept the same as the parameters that are listed in Table 1. Since the structure is symmetric, it is sufficient to only show the S_11_ and S_21_. Figure 5 shows the effects of *r* (outer radius of the main resonator) on the S-parameters. It can be observed that as L increases, the lower frequency bandwidth shifts down slowly to a lower band and also the upper operation frequency moves fast toward a lower band, which means that the length of the slot radiator changes its capacitance characteristic [39,40]. As seen, by changing the size of r from 12 to 16 mm, the lower and upper frequencies of the antennas move from 3.5 to 1.5 GHz and 11 to 8 GHz, respectively. In addition, by changing the values of r, the mutual coupling (S_21_) of the design is changed, especially at middle frequencies, as illustrated in Figure 5b. 

Figure 6 and Figure 7 represent the impact of *L* (length of arc-shaped radiation patch) and *r_1_* (inner radius of the slot resonator) on the S-parameters of the designed UWB dual-polarized antenna. It is found that, unlike the outer radius of the main resonator (r), L and r_1_ mainly affect the lower operation band of the antenna. As shown in Figure 6a, as L decreases from 12 to 6 mm, the lower frequency bandwidth changes from 2 to 4 GHz. It should be noted that changing the values of L does not significantly impact the transmission coefficients (or mutual coupling) that are characteristic of the antenna (shown in Figure 6b). Figure 7 illustrates the simulated S-parameter results of the antenna for various values (9 to 7 mm) of the inner radius (r_1_) of the slot resonator. It is clear that, when the inner radius of the slot varies from 9 to 7 mm, the lower operation bandwidth of the design tunes from 2.1 to 3.1 GHz. In addition, this parameter value changing also affects the isolation of the mutual coupling characteristic of the design, as illustrated in Figure 7b. It is evident that, by the reduction of the inner radius of the slot, the isolation of S_21_ has been significantly improved.

**Figure 9:**
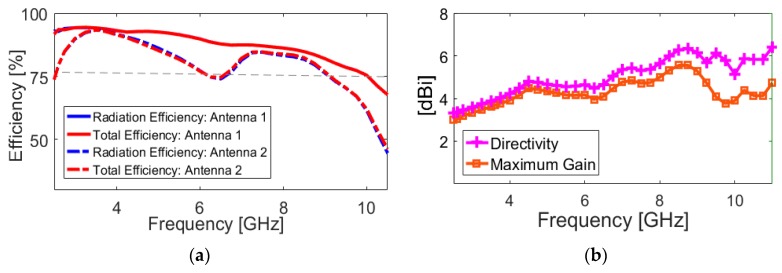
(**a**) Radiation/total efficiencies, maximum gain and (**b**) diversity results of the diversity antenna.

The effects of another important design parameter, the employed rotated strip on the slot resonator (W_1_), on antenna performance are investigated through simulation, as presented in Figure 8. It can be found from Figure 8a that the isolation of the resonant bands at lower and upper frequencies changes for different values of W_1_. Meanwhile, the change of the mutual coupling with the variation of is W_1_ is not significant. Figure 9a provides the simulated efficiencies (radiation and total) of the diversity antenna design. As shown from the figure that more than 75% radiation efficiency is obtained over the ultra-wide impedance bandwidth of the antenna with the maximum value of 90% at the lower frequencies. This is caused by the enhanced isolation characteristic of the antenna. Moreover, it is evident that the UWB antenna exhibits good total efficiency results of 50%~75%. Figure 9b depicts the maximum gain and directivity results versus the investigated frequency range. It is shown that, although the gain varies with the operation frequency, its level is still more than 3.1 dBi within the frequency band of interest. It is seen that the difference between directivity and maximum gain results is small, which is mainly because of the high-efficiency characteristic of the antenna. The overall variation of the gain and directivity is within 3 dBi in the entire frequency band, which is very good for wideband and multiband systems. 

In the UWB antenna system, the time-domain characteristics are equally as important as the frequency domain. In the time domain method, an important factor indicating the characteristic of the antenna is the fidelity factor. The values of the system fidelity factor vary between 0 and 100%. A system fidelity factor value of 100% shows that the received signal perfectly fits the input signal [41]. The fidelity is employed as a factor of similarity between the input and received signal and is obtained, as follows:

(1) $$F=Max_{\tau }\left|\frac{\int_{-\infty }^{+\infty }s\left(t\right)r\left(t-\tau \right)}{\sqrt{\int_{-\infty }^{+\infty }s{\left(t\right)}^{2}dt.\int_{-\infty }^{+\infty }r{\left(t\right)}^{2}dt}}\right|$$

where *s(t)* and *r(t)* are the input and received signals, respectively. Two identical configurations of the dual-polarized UWB antenna, including side-by-side and face-to-face orientations with a 100 mm shift of their center points, are studied. The antenna is excited by using a modulated Gaussian pulse. 

**Figure 10:**
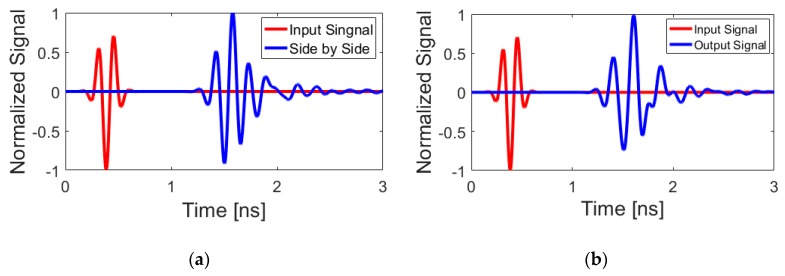
Input/output signal pulses for (**a**) side by side and (**b**) face to face scenarios.

**Figure 11:**
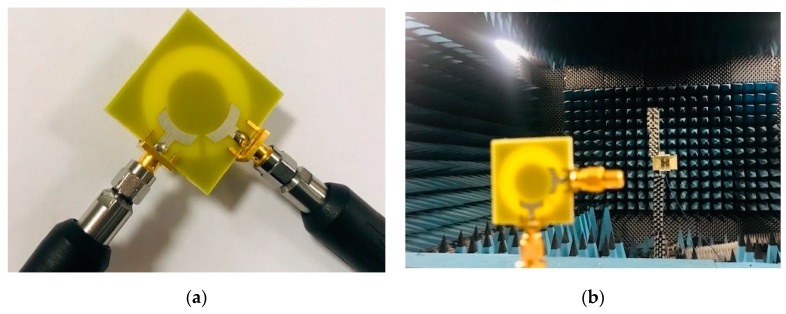
Photographs of the antenna in measurements setup of (**a**) S-parameters and (**b**) radiation patterns.

**Figure 12:**
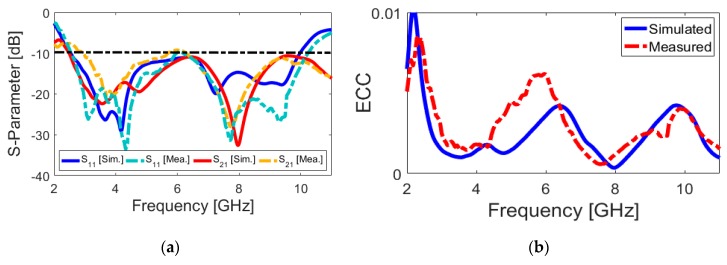
Measured and simulated (**a**) S-parameters and (**b**) ECC results of the proposed antenna.

A relatively good similarity has been achieved for the *R_X_* and *T_X_* pulses, as shown in Figure 10. In addition, using (1) the fidelity factor of the reference antenna pair are calculated and good results have been obtained (equal to 0.85 and 0.75, respectively). A prototype sample of the proposed UWB dual-polarized antenna is fabricated and tested. Figure 11 shows the placements of the fabricated prototype in measurement setups of S-parameters and radiation patterns, respectively. The measured S-parameters (S_11_&S_21_) of the fabricated sample is examined while using the vector network analyzer and illustrated in Figure 12a. It is found that the fabricated prototype works properly and it provides acceptable agreement with the simulations. As shown, a good frequency bandwidth (S_11_ ≤ −10 dB) of 2.5–10 GHz is achieved for the fabricated dual-polarized UWB antenna. However, for S_11_ ≤ −6 dB, this value could be from 2.2–11 GHz. In addition, it is evident that the mutual-coupling characteristic of the design is less than −10 dB over the entire UWB operation frequency band.

In order to validate the capability of a dual-polarized MIMO antenna design, envelope correlation coefficient (ECC), total active reflection coefficient (TARC), and its diversity gain (DG) properties are three important parameters to be investigated [42,43]. The acceptable limits of standards are ECC < 0.5, TARC < −10, and DG near 10 dB. These characteristics of MIMO antenna can be calculated from the S-parameter results while using the below formula:

(2) $$ECC=\frac{{\left|S_{mm}^{*}S_{mn}+S_{nm}^{*}S_{nn}\right|}^{2}}{\left(1-{\left|S_{mm}\right|}^{2}-{\left|S_{mn}\right|}^{2}\right){\left(1-{\left|S_{nm}\right|}^{2}-{\left|S_{nn}\right|}^{2}\right)}^{*}}$$

(3) $$TARC=-\sqrt{\frac{{\left(S_{mm}+S_{mn}\right)}^{2}+{\left(S_{nm}+S_{nn}\right)}^{2}}{2}}\;$$

(4) $$DG=10\sqrt{1-{\left(ECC\right)}^{2}}$$

**Figure 13:**
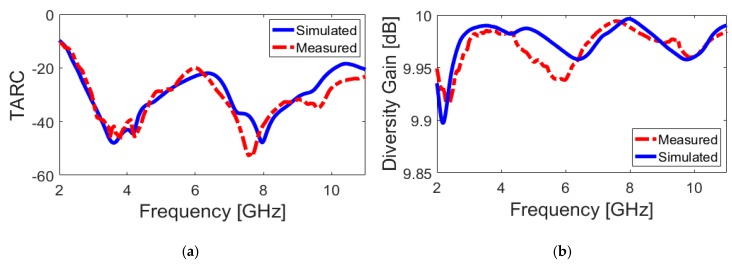
Calculated (**a**) TARC and (**b**) DG results from the measured and simulated results.

Figure 12b illustrates the calculated ECC characteristic of the antenna. It can be observed that the calculated ECC results are very low entire the UWB operation band (less than 0.01). Figure 13a,b illustrate the TARC and DG characteristics of the antenna, respectively. It is also found in Figure 13a that the TARC value of the dual-polarized UWB-MIMO design is less than −20 dB in the frequency range of 2.5–10 GHz. The diversity gain function of the designed antenna over its operation band is more than 9.95 dB over the entire operating frequency band, as can be seen in Figure 13b.

**Figure 14:**
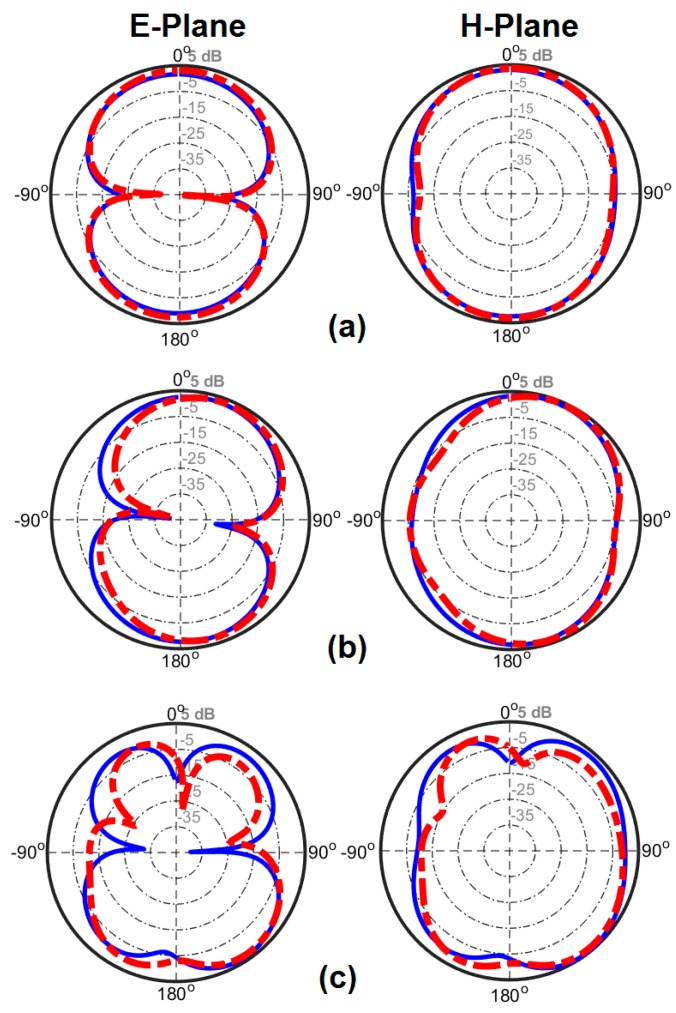
Measured (dash line ) and simulated (solid line) results of the antenna patterns at (**a**) 3 GHz, **(b**) 6 GHz, and (**c**) 9 GHz.

In addition, two-dimensional (2D) radiation patterns of the fabricated antenna have been measured. Figure 14 shows the measured/simulated radiation patterns of the antenna at selected frequencies, including 3, 6, and 9 GHz. These three frequencies are chosen form the lower, middle, and upper frequencies, respectively. In this design, the xz plane is H-plane (φ = 0°) and yz-plane is E-plane (φ = 90°) for the proposed antenna. From Figure 14, we can see that the antenna can gives dumbbell-like radiation characteristics in E-plane and nearly Omni-directional patterns in H-plane [44,45]. It was found that the radiation patterns of the UWB antenna deteriorate more or less with increasing frequency. However, the radiation properties are almost stable. 

**Figure 15:**
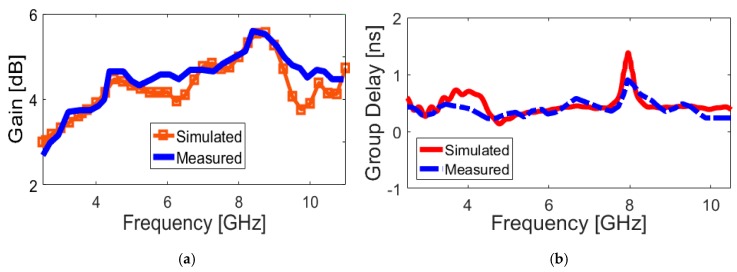
Measured and simulated (**a**) antenna gain and (**b**) group delay over its ultra wide bandwidth.

The peak gains of the UWB dual-polarized antenna over its operation band are measured and compared with simulations, as illustrated in Figure 15a. Almost stable constant gain values are achieved over the antenna operation band, as can be observed. However, the antenna gain is increased from 3.2 to nearly 5.8 dB, which is caused by the deteriorated radiation at the higher band. As a well-known fact, a UWB antenna should cover a wide band. Therefore, the analysis of group delay is very important [46]. When a signal goes through a filter, the signal will distort in both amplitude and phase. This distortion depends on the characteristics of the designed filter, which can determine the communication quality. By representing the transmitting and receiving antennas as a filter, the group delay at the operation band is very important for designing UWB antennas. The group delay is defined as the measurement of the signal transition time through a device. For the UWB antennas, the altered inductance properties result in a group delay of UWB antennas. The group delay characteristic can be defined through the derivative of phase, which is expressed as:

(5) $$\tau \left(\omega \right)=-\frac{\partial \varphi \left(\omega \right)}{\partial \left(\omega \right)}\;$$

where φ(ω) and ω are the phase and angular frequencies, respectively. Figure 15b illustrates the measured/simulated group delay of the designed UWB antenna. The result shows that the group delay variation is less than 1 ns in the entire operation band. This indicates a linear phase response and good pulse handling capability for the proposed antenna. Hence, the antenna is useful in the UWB impulse radio and microwave imaging.

Table 2 provides a comparative summary of the fundamental properties of the presented design with the reported dual-polarized antennas available in the literature [47,48,49,50]. When compared with reported designs, the presented antenna has a simple structure with a compact, as summarized in Table 2. In addition, a wider impedance bandwidth providing more than 120% FBW is achieved for the proposed antenna. As shown, in contrast with the reported designs in the literature, the proposed dual-port diversity antenna exhibits lower ECC results. Stable and almost constant gain values are achieved for the antenna over the antenna UWB operation band, which is very good for wideband and multiband systems. Furthermore, as explained earlier, high radiation efficiencies are achieved for the proposed design. Therefore, the antenna is suitable for different wireless applications, such as radar, microwave imaging, and cellular communications, due to these attractive features.

## 3. The Proposed UWB Diversity Antenna System

**Figure 16:**
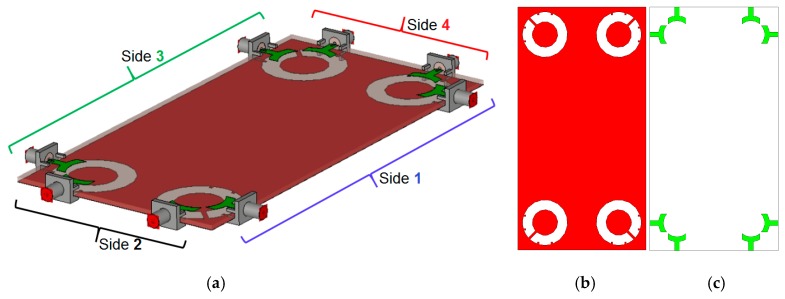
(**a**) Side, (**b**) top, and (**c**) bottom views of the UWB-multiple-input/multiple-output (UWB-MIMO) antenna system design for smartphone applications.

Figure 16 shows the schematic of the MIMO mobile-phone antenna system. As shown, it comprises four identical pairs of compact microstrip-fed slot antennas with polarization diversity function that are placed symmetrically at different edge corners of the mainboard with a standard dimension of 75 × 150 × 1.6 mm^3^. However, it is also possible to arrange the design in different sizes of the handset mainboard, due to the compact sizes of the employed diversity slot elements.

**Figure 17:**
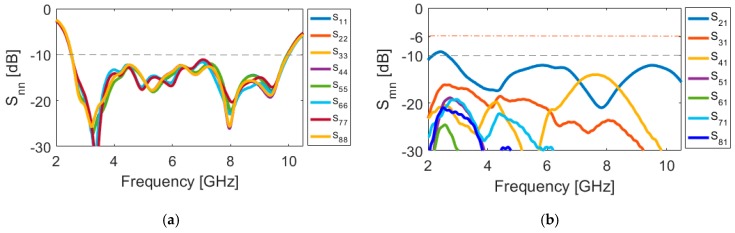
(**a**) S_nn_ and (**b**) S_mn_ characteristics of the simulated MIMO design.

Figure 17 depicts the S parameters (including S_nn_ and S_mn_). As shown, the antenna exhibits good S parameters, similar to the single-element diversity antenna, covering the frequency spectrum of 2.5–10 GHz. In addition, the mutual coupling of the design is below −10 over the operation frequency of the smartphone antenna, which meets the basic requirements for MIMO operation. As expected, the maximum mutual coupling of the MIMO design is between the closely spaced diversity elements (S_21_ (port 1 and port 2, for example)) with a fixed size of the antenna element (W_S_ × L_S_). In addition, the mutual coupling characteristics of other port pairs is less than −16 dB. This function makes the design flexible to be arranged in different sizes of PCB. Therefore, it is possible to get satisfactory results for smaller motherboard dimensions.

Using (1), the fidelity factor of the MIMO antenna design is also investigated. In the proposed MIMO smartphone antenna array, the fidelity factor is the highest between the closely-spaced diversity antenna pairs with the side-by-side orientation, averaging 0.90. It should be noted that the fidelity factor can be decreased as the distance increases and vice versa [57]. Accordingly, for other possible arrangements of the antenna pairs with longer distances, the fidelity factor drops to 0.70 in most cases. However, the value is still reasonably good and acceptably low. 

**Figure 18:**
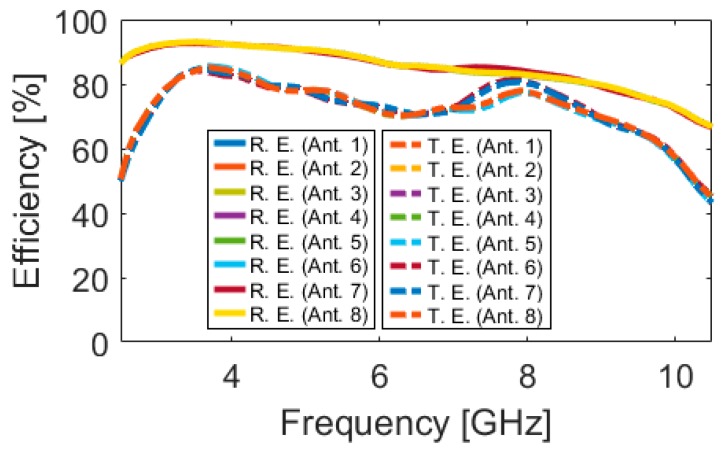
Efficiencies of the MIMO design.

**Figure 19:**
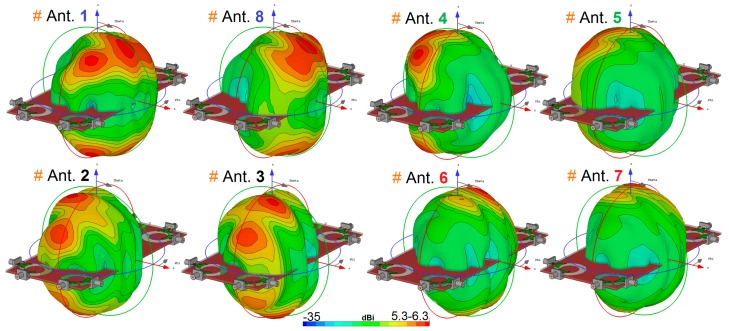
Radiation patterns of the diversity antenna element with directivity value at 5.5 GHz.

Figure 18 illustrates the radiation efficiency (R.E.) and total efficiency (T.E.) properties of the proposed design. According to the results, it can be concluded that the MIMO design provides sufficient efficiencies over its entire operation band [58,59]. Three-dimensional (3D) radiation patterns of each element at 6 GHz (middle frequency) have been plotted in Figure 19. It should be noted that the shapes of the radiation patterns for the antenna elements are a bit different from single-element, which makes it more suitable for the smartphone application. The radiation patterns of the smartphone antenna not only can cover different sides of the smartphone board, but also generate different polarizations, which is a unique function for MIMO design, as mentioned above [60]. This is mainly due to the corner placement of the antenna pairs as well as the big ground plane of the MIMO design. As can be observed from Figure 19, different sides (including sides 1, 2, 3, and 4, as shown in Figure 16a) of the PCB board are covered by different elements with different polarization. Therefore, in total, the designed UWB smartphone antenna contains four horizontally-polarized and four vertically-polarized radiation elements. 

**Figure 20:**
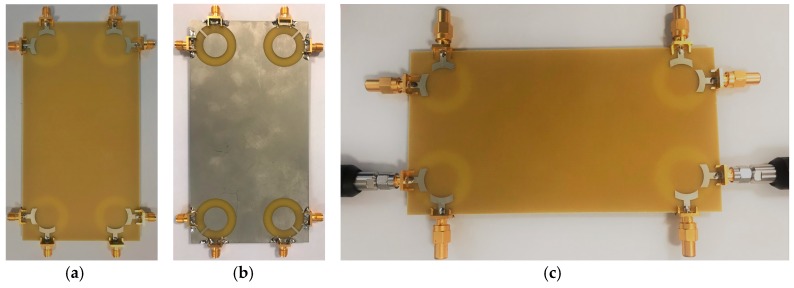
Fabricated prototype, (**a**) top view, (**b**) bottom view, and (**c**) feeding mechanism.

**Figure 21:**
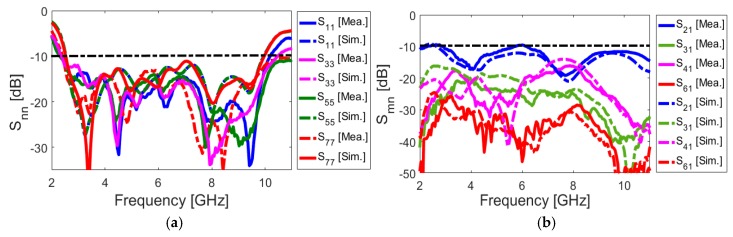
Measured and simulated (**a**) S_nn_ and (**b**) S_mn_ characteristics.

The proposed UWB dual-polarized antenna system was fabricated and its S-parameters, radiation patterns, and gain characteristics are measured. Figure 20a,b show the top and bottom layers of the prototype. The MIMO smartphone antenna is implemented on a cheap FR4 substrate with an overall size of 75 × 150 × 1.6 mm^3^. Figure 20c illustrates the measurement setup of the fabricated antenna. As can be observed, 50-Ohm loads are employed for the elements, not under measurement in order to measure the characteristics of the MIMO system and also to eliminate the mutual effects from the other elements. Figure 21a,b depict the simulated and measured S_nn_ and S_mn_ results of the MIMO design, respectively. It should be noted that, due to the similar behavior of the elements, it is not necessary to show all of the achieved S-parameters. Similar S_nn_ results with ultra-wide bandwidth are obtained for the antenna elements, as shown in Figure 21a. In addition, all S_mn_ results of the design are less than −10 dB. Good agreement is observed for the presented MIMO smartphone antenna when compared with the simulations. 

**Figure 22:**
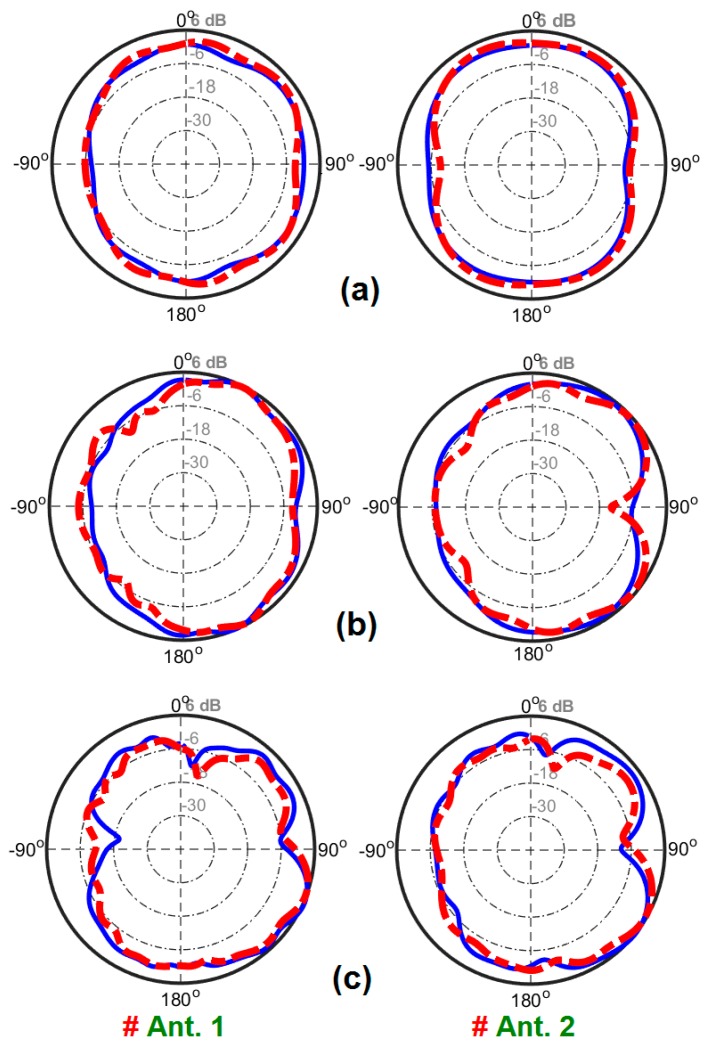
Measured (dash line ) and simulated (solid line) radiation pattern results for Ant. 1 and Ant. 2 at (**a**) 3 GHz, (**b**) 6 GHz, and (**c**) 9 GHz.

The 2D-polar radiation patterns of two adjacent elements (including Ant. 1 and Ant. 2) are measured and compared with the simulations due to similar performances of the radiation elements of the smartphone antenna. Figure 22 plots the measured and simulated radiation patterns of the antennas at 3, 6, and 9 GHz. It is found that the fabricated smartphone antenna can provide quasi-omnidirectional radiation patterns at different frequencies of its operation bandwidth [61]. The experimental and simulation results agree well with each other, as can be observed. Next, we evaluate the TARC and ECC characteristics of the proposed UWB MIMO smartphone antenna. Since these two parameters consider the mutual effects, they can provide more meaningful measures of the designed MIMO antenna performance than the reflection coefficient. 

**Figure 23:**
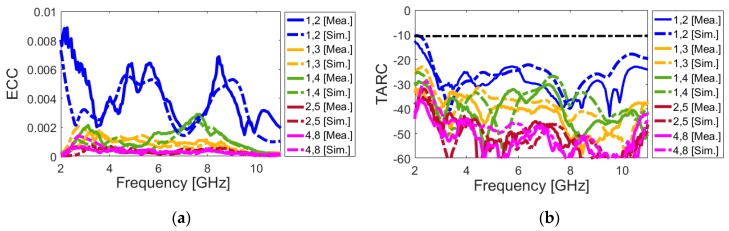
Calculated (**a**) ECC and (**b**) TARC results of the UWB smartphone antenna from measured/simulated results.

Figure 23a,b depict the ECC and TARC parameters, as calculated from the measured/simulated results. It can be observed from Figure 23a that the designed MIMO antenna provides very low ECC (less than 0.01) through its entire operation band. Furthermore, it is clear from Figure 23b that the UWB MIMO antenna design exhibits low TARC characteristics over the ultra-wide bandwidth (2.5–10 GHz). As seen, the design has less than −20 dB TARC values at different frequencies. The fundamental properties of the proposed MIMO diversity antenna system are compared with the recently reported MIMO antenna designs and are listed in Table 3 [21,22,23,24,25,26,27,28,29,30,31,32,33,34,35,36,37,38]. As clearly seen, in contrast to the recently proposed designs, the designed MIMO antenna offers ultra-wide frequency bandwidth of 2.6–10 GHz (more than 120% FBW), a unique characteristic that none of the other MIMO antenna designs cited have. In addition, it can be seen that the presented MIMO antenna exhibits higher performances in terms of efficiency, gain level, ECC, and TARC characteristics. Furthermore, the proposed MIMO smartphone antenna exhibits radiation pattern diversity and also supports different polarizations (including vertical/horizontal) at different four sides of the mainboard, unlike most of the reported MIMO designs.

Another important function is the channel capacity loss (CCL) of the MIMO system [43], which mainly depends on the S-parameters of the MIMO system with the accepted limit of ≤0.4 bps/Hz and can be calculated while using the below formulas:

(6) $$CCL=-log_{2}\det\left(\psi^{R}\right)$$

(7) $$\psi^{R}=\left[\begin{array}{ccc} \rho_{11} & \cdots & \rho_{18}\\ \vdots & \ddots & \vdots \\ \rho_{81} & \cdots & \rho_{88} \end{array}\right]$$

where,

$\rho_{ii}=1-\left({\left|S_{ii}\right|}^{2}+{\left|S_{ij}\right|}^{2}\right)$
$\rho_{ij}=-\left(S_{ii}^{*}S_{ij}+S_{ji}^{*}S_{ij}\right)$ for *i,j* = 1,…,8

**Figure 24:**
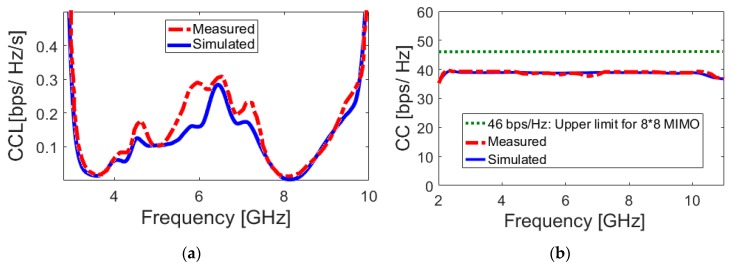
Calculated (**a**) channel capacity loss (CCL) and (**b**) channel capacity (CC) of the smartphone antenna.

Figure 24a illustrates the calculated channel capacity loss from the measured and simulated results of the proposed UWB-MIMO smartphone antenna. It is shown that the MIMO antenna design offers a very low CCL band: less than 0.3 bps/Hz over the operation band of 2.7~10 GHz is obtained. Furthermore, the calculated channel capacity (CC) is investigated in Figure 24b in order to further study the MIMO operation of the presented UWB antenna system. The CC is defined as the maximum possible transmission rate, such that the probability of error is arbitrarily small. It is defined as:

(8) $$CC=E\left\{log_{2}\left[det\left(I+\frac{SNR}{n_{T}}\right)H_{scale}H_{scale}^{T}\right]\right\}$$

where the channel matrix *H_scale_* can be calculated using:

(9) $$H_{scale}=\sqrt{\rho_{scale,RX}\;}H_{i.i.d}\;\sqrt{\rho_{scale,TX}}$$

As shown, the calculated CC within the entire operating frequency is around 40 bps/Hz, whereas, for the ideal case, it is about 46 bps/Hz [62]. According to the obtained ECC, TARC, and CCL results, it can be calculated that the proposed UWB smartphone antenna has provided worthy MIMO performance.

## 4. Conclusions

A new design structure of eight-port UWB-MIMO mobile-phone antenna is proposed for future mobile handsets. Its configuration consists of four pairs of diversity slot antenna elements located at four edge corners of the PCB mainboard with an FR-4 dielectric. The circular-ring slot radiator is converted to an open-ended slot by adding a rectangular strip in order to enhance the port isolation and increase the impedance-matching of the closely-spaced ports. The antenna elements exhibit ultra-wide impedance bandwidth, covering 2.6–10 GHz (S_11_ ≤ −10 dB), and provide radiation pattern and polarization diversity function. Acceptable fundamental properties in terms of input-impedance, antenna gain, efficiency, radiation patterns, and user-effect are achieved for the proposed design. Low ECC, TARC, DG, and CCL characteristics have been obtained. Moreover, the performance of the designed UWB-MIMO smartphone antenna for different user-hand/user-head scenarios has been studied and sufficient efficiency performances are obtained. The antenna has a simple and planar structure under the premise of covering different frequencies, which makes it suitable for MIMO operation in future smartphone applications.

## Figures and Tables

**Table 1:** Parameter values of the proposed UWB diversity designs.

Parameter	W_S_	L_S_	h_S_	W_f_	L_f_	d
**Value (mm)**	34	34	1.6	5.5	3	7.5
**Parameter**	h_S_	W	L	W_1_	L_1_	r
**Value (mm)**	1.6	1	11	1.5	1.5	11.5
**Parameter**	r_1_	r_2_	r_3_	W_sub_	L_sub_	H_sub_
**Value (mm)**	8.5	8	14	75	150	1.6

**Table 2:** Comparison between the Design and the Recently Reported Dual-Polarized UWB Antennas.

Reference	FBW (%)	Size (mm^2^)	Gain (dB)	ECC
[47]	115% (2.9–11 GHz)	105 × 105	2–7	-
[48]	112% (3–11.5 GHz)	90 × 90	4–9	-
[49]	120% (3–12 GHz)	76.25 × 52.25	4–6	<0.05
[50]	107% (3–10 GHz)	72 × 72	3–6	-
[51]	120% (3–12 GHz)	66.25 × 66.25	5	<0.1
[52]	112% (3.1–11.8 GHz)	61 × 68	1–7	<0.02
[53]	114% (3–11 GHz)	57 × 57	5	<0.02
[54]	114% (3–11 GHz)	56 × 56	3–5	<0.02
[55]	120% (3–12 GHz)	40.5 × 40.5	5	<0.1
[56]	114% (3–11 GHz)	35 × 35	4.6	<0.3
Proposed Antenna	121% (2.5–10.2 GHz)	34 × 34	4–6	<0.01

**Table 3:** Comparison between the Proposed Smartphone Antenna and the Reported MIMO Antennas.

Ref.	Design Type	B.W. (GHz)	Efficiency (%)	Size (mm^2^)	ECC
[21]	Inverted-F	3.4–3.6	-	120 × 70	-
[22]	Monopole	3.4–3.6	35–50	150 × 75	<0.40
[23]	Tightly Arranged Pairs	3.4–3.6	50–70	150 × 73	<0.07
[24]	Integrated Waveguide	3.4–3.6	50–80	150 × 75	<0.2
[25]	Modified PIFA	3.25–3.85	40–75	150 × 75	<0.01
[26]	Loop	2.55–2.6	48–63	136 × 68	<0.15
[27]	SCS patch-slot	3.55–3.65	52–76	150 × 75	-
[28]	Monopole-Slot	2.55–2.68	48–63	136 × 68	<0.15
[29]	Slot	3.4–3.8	50–75	150 × 75	<0.01
[30]	circular-slot loop	3.3–3.9	60–80	150 × 75	<0.01
[31]	Monopole	4.55–4.75	50–70	136 × 68	-
[32]	Inverted-F	3.4–3.6	55–60	100 × 50	-
[33]	Self-Isolated Monopole	3.4–3.6	60–70	150 × 75	<0.015
[34]	Inverted-L Monopole	3.3–5	40–60	136 × 68	<0.2
[35]	Identical Monopole	2–6	30–60	124 × 64	-
[36]	U-Slot	3.3–6	40–75	150 × 75	<0.12
[37]	Loop	3.3–5	40–70	150 × 75	<0.1
[38]	Inverted-F	3.3–4.5	20–75	150 × 75	<0.6
This Work	Diversity Ring-Slot	2.6–10.2 (122%)	60–80	150 × 75	<0.007
